# A 26-Year-Old Retained Demised Abdominal Pregnancy Presenting with Umbilical Fistula

**DOI:** 10.1155/2014/932525

**Published:** 2014-02-03

**Authors:** Nnadi Daniel, Bello Bashir, Ango Ibrahim, Singh Swati

**Affiliations:** ^1^Department of Obstetrics & Gynaecology, Usmanu Dan-Fodio University Teaching Hospital (UDUTH), PMB 2370, Sokoto, Nigeria; ^2^Department of Surgery, Usmanu Dan-Fodio University Teaching Hospital (UDUTH), Sokoto, Nigeria

## Abstract

This is a report on a 72-year-old postmenopausal woman who presented with passage of fetal bones through an umbilical fistula. She was diagnosed as a case of demised abdominal pregnancy, which had been retained for 26 years. She subsequently had exploratory laparotomy, evacuation of the abdominal pregnancy, hysterectomy, and bowel resection. The patient's condition remained unstable throughout the postoperative period and she died from septicemia on the eleventh day.

## 1. Introduction

**Figure 1:**
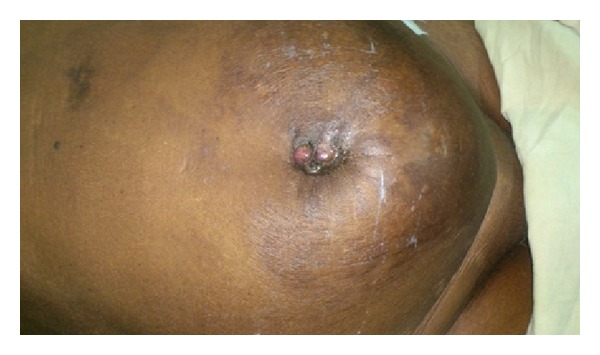
Umbilical fistula.

Abdominal pregnancy is a rare form of ectopic pregnancy where the conceptus implants in the abdominal cavity [1]. This is mostly a result of reimplantation of ruptured undiagnosed tubal ectopic pregnancy [2]. The highest incidence in the world is found among the South African Bantu tribes, where it accounts for 2.2% of all ectopic pregnancies [3]. In Sokoto, Nigeria, an incidence of 3.1/10,000 deliveries has been reported, while in the United States of America (USA), it occurs in 1 in 3372 to 7931 pregnancies [4, 5]. Diagnosis of abdominal pregnancy presents a dilemma to many physicians, because the presenting symptoms are variable. A high index of suspicion is the cornerstone to diagnosis and early diagnosis is necessary to improve prognosis. Maternal mortality is very high and it is usually due to severe intraabdominal hemorrhage and sepsis [4]. The fetal prognosis is also very poor due to friability and poor vascularity of the placental implantation site, resulting in fetal growth restriction and fetal anomalies [4]. In situations where the fetus has demised, it may be retained and undergo mummification particularly when the pregnancy is advanced and abdominal pregnancy has been undiagnosed. We present a case of a 72-year-old postmenopausal woman who presented with passage of fetal bones through an umbilical fistula as a result of a demised abdominal pregnancy which had been retained for 26 years (Figure 1).

## 2. Case Report

**Figure 2:**
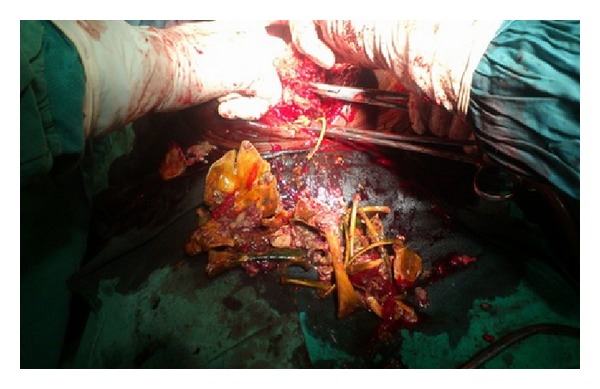
Intra operative evacuation of fetal bones.

**Figure 3:**
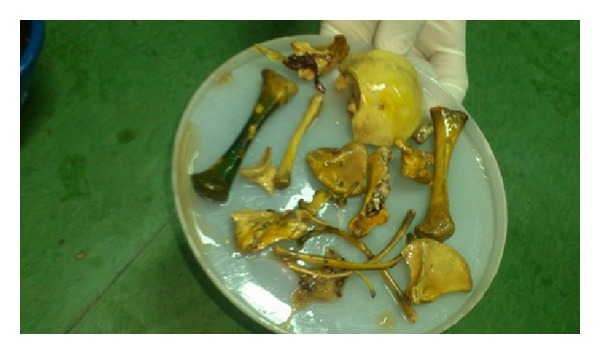
The evacuated fetal bones.

The patient was a 72-year-old Para 4A2 widow from Sokoto, Nigeria, who was 26 years postmenopausal. She was referred from the surgical outpatient clinic (SOPD), with a 2-week history of passage of bony substances suspected to be fetal bones through the umbilicus (Figures 2 and 3). There was a preceding one-year history of purulent discharge from the umbilicus. The discharge was occasionally bloody and she noticed three pieces of bone drop spontaneously from the discharging point. There was no antecedent trauma or surgical procedures on the abdomen. She had no history of vaginal bleeding or discharge. She was pregnant before the death of her husband about 27 years ago. The pregnancy was confirmed by a transabdominal ultrasound and she booked for antenatal care at specialist hospital, Sokoto. She discontinued her visits when fetal movements suddenly ceased and the abdominal girth progressively regressed. The pregnancy had remained undelivered ever since.

The patient was a Para 4A2, all females. Her 1st two children died at 3 and 9 years of age, respectively. Previous pregnancies were not booked for antenatal care and all her deliveries were unsupervised. She had no knowledge of contraceptives and had never used any form. She had not been sexually active since the demise of her spouse. Her past medical history was not significant. She was a pauper and was not formally educated.

Physical examination revealed an elderly woman who was moderately pale and had pedal and periorbital edema. She was anicteric and afebrile to touch. The pulse rate was 108 beats per minute and the blood pressure was 110/70 mmHg. She weighed 47 kg. The chest was clinically clear and heart sounds were normal. The abdomen was distended with indurations in the periumbilical region but moved with respiration. The umbilical orifice was discharging a foul smelling purulent material. There were 3 human bones determined to be the fetal tibia and humerus presented by the patient. There was a hard, fixed pelvic mass of about 24 weeks size with irregular contours. The mass had both cystic and solid components. There was tenderness around both iliac fossae. The liver, spleen, and kidneys were not palpably enlarged. Pelvic examination revealed an atrophic vulva and vagina. The cervix was flushed to the upper part of the vagina and the cervical Os was closed. There was difficulty in moving the cervix and the patient declined further examination. A diagnosis of umbilical bony fistula following a neglected demised abdominal pregnancy to rule out ovarian germ cell tumour was made. General and specific investigations were requested. The PCV was 21%, WBC 6.8 × 10^9^/L, and the platelet count was 226 × 10^9^/L. The liver and renal function tests all showed normal values. A plain abdominal X-ray revealed abdominal distension with multiple air fluid levels. The bowel loops, caliber, and distribution were normal. There was an irregularly outlined mass of calcific density projected over the pelvis, measuring about 9 cm × 9 cm. A sharply defined continuous structure was seen holding the mass. The underlying bone showed moderate osteoporosis and spondylosis. The impression was a huge bony calcific mass in the pelvis. A transabdominal ultrasound showed multiple calcifications in the pelvis, the size of which could not be measured. A CT-scan revealed a grossly calcified amorphous intraperitoneal tumor with adherence to the anterior abdominal wall and umbilicus. The mass measured 93 mm × 8.7 mm. There were matted omental and mesenteric tissues. The uterus was atrophic and it was difficult to delineate the adnexae.

A wound swab was taken for microscopy and culture. She received intravenous antibiotics and oral anti-inflammatory drugs. Daily dressing of the umbilical fistula with EUSOL and hydrogen peroxide was instituted. She was transfused with 3 units of blood and had a review from the general surgeons. The patient was counseled and the social welfare department of the hospital accepted to foot her hospital bills. She was prepared for exploratory laparotomy. On 25/7/2013, the patient was taken to the theatre and had exploratory laparotomy, delivery of abdominal pregnancy, subtotal hysterectomy, adhesiolysis, bowel resection, and end-to-end anastomosis of the resected bowel segment, under general anesthesia.

The intraoperative findings were a longitudinal uterine rent in the posterior uterine wall, extending to the fundus uterine. There was a well-formed fetal skeleton. There were protrusions of bony tissues—mainly small bones of the upper limbs, skull, the ribs, and spinal cord. It was difficult to visualize the uterine tubes, ovaries, and bladder due to dense adhesions. There were massive bowel adhesions and a damaged segment of the ileum, about 20 cm from the ileo-cecal junction. Adhesiolysis was done with difficulty and the damaged segment was resected and end-to-end anastomosis was performed. Subtotal hysterectomy was performed following the standard procedure.

The estimated blood loss was 1,200 mL. The procedure was well tolerated by the patient, she had 2 units of blood intraoperatively, and 3 units were transfused postoperatively. Parenteral antibiotics were also given but the vital signs remained unstable. The patient died on the 11th postoperative day. The cause of death was septicemia.

## 3. Discussion

Abdominal pregnancy, though a rare condition, continues to generate interest because it is associated with high maternal and perinatal morbidity and mortality [6]. The incidence is influenced by the frequency of ectopic gestation in the population, socioeconomic status of the patient, the degree of utilization of medical care in the country, and the level of medical care available in the institution [7]. Incidence is thus higher in resource-poor countries. The common risk factors include infertility, previous pelvic infection, congenital anomalies of the uterine tubes, endometriosis, and previous ectopic pregnancy [4, 8]. This patient's poor socioeconomic status and hence poor health-seeking behaviour may have led to the late presentation and unfavorable outcome of this case. Because of comparatively few symptoms, abdominal pregnancy is often recognized late. It remains a diagnostic and therapeutic challenge for every obstetrician. Thus, an obstetrician needs to have a high index of suspicion in order to prevent the related complications.

The clinical presentation of abdominal pregnancy depends on gestational age. In the first trimester, the symptoms are similar to those of tubal pregnancy. Thus, recent advances in imaging techniques such as ultrasound, magnetic resonant imaging (MRI) CT-scan, and immunochemistry have aided in some measures in the early recognition of abdominal pregnancy in the first trimester, but advanced abdominal pregnancy is a rare event and presents a diagnostic challenge. The diagnosis was made possible in the index case because of the clinical symptoms of persistent lower abdominal pain, suprapubic mass, and passage of fetal bones through a discharging umbilical sinus and also with the aid of imaging technology. In a 10-year review of abdominal pregnancy in Sokoto, Nigeria, Nnadi et al. observed that in most cases, persistent lower abdominal pain was likely to be present [4].

If an abdominal pregnancy is neither diagnosed nor treated and if the fetus dies intraabdominally, the end result may be a calcified product of conception known as lithopedion. Intraabdominal calcification instead of spontaneous reabsorption of a dead mummified fetus is an unusual complication of abdominal pregnancy. Fistula formation is common in situations where the pregnancy is advanced, demised, undiagnosed, and retained for a very long time. This was the situation in this case where the pregnancy has been retained for 26 years. There was a reported case of lithopedion that had been retained for 29 years [8, 9]. Fistula formation may result from penetration of fetal bones into maternal bowel, rectum, vagina, bladder, or abdominal wall, as was observed in this patient. Undiagnosed abdominal pregnancy presenting as faecal fistula has been reported from Ile-Ife, Nigeria [10]. The earliest descriptions of abdominal pregnancies presenting with fistula formation were recorded about 1000 years ago, when Abdulcasis observed the discharge of fetal parts through the abdominal wall in the umbilical region [8, 9]. Further umbilical fistulae discharging fetal parts were described in the 16th century by Conax in 1545, Felix Platter in 1584, and Jacob Nolerus in 1595 [8].

Pitfalls in establishing diagnosis by ultrasound have been described [7]. The treatment of advanced abdominal pregnancy is associated with an enormous risk of life-threatening maternal hemorrhage [11]. The risk of dying from abdominal pregnancy is 7.7 times greater than from a tubal pregnancy and 90 times higher than from intrauterine pregnancy [3]. Thus, careful preoperative preparation including adequate supply of compatible blood and blood products should be made. This patient despite the necessary cautions taken still died after five days of surgery. Her advanced age and presence of comorbidities may have been contributing factors.

## 4. Conclusion

Advanced abdominal pregnancy retained for 26 years in a 72-year-old postmenopausal woman is a very rare event. The patient's ignorance and poor utilization of medical services were contributing factors. Late presentation, prolonged retention of dead mummified fetus, extensive bowel adhesions, and advanced maternal age lead to unfavorable outcome.
